# Isolated brachioradialis metastasis of gastric adenocarcinoma after R0 resection

**DOI:** 10.1186/s12957-021-02191-9

**Published:** 2021-03-20

**Authors:** Elizabeth Jacob, Levi Smucker, Ryan Crouse, Ayana Allard-Picou

**Affiliations:** 1 Department of Surgery, Bassett Medical Center, Cooperstown, NY USA; 2 Columbia University College of Physicians and Surgeons, New York City, USA

**Keywords:** Gastric cancer, Metastatic cancer, Tumor biology, D2 lymphadenectomy

## Abstract

**Background:**

Gastric cancer is the fifth most common cancer worldwide, with an incidence of 6.72 per 100,000 people. Thirty-two percent of gastric cancer patients will live 5 years after diagnosis. Single-site metastasis is noted in 26% of patients with gastric cancer, most commonly in the liver (48%), peritoneum (32%), lung (15%), and bone (12%). Here, a case is presented in which a single skeletal muscle metastasis appeared after appropriate resection and treatment.

**Case presentation:**

A 63-year-old man underwent neoadjuvant chemotherapy and a multivisceral en bloc R0 resection. Final pathology showed no evidence of lymph node metastasis with 31 negative lymph nodes. Four months postoperatively, the patient was found to have a rapidly growing biopsy-proven extremity soft tissue gastric metastasis within the brachioradialis muscle. He subsequently underwent metastasectomy and immunotherapy.

**Conclusion:**

This case is a rare example of an isolated extremity metastasis of gastric adenocarcinoma in the setting of an R0 resection of the primary tumor and negative nodal disease on final pathology, suggestive of hematogenous spread. We review the biology, workup, and management of gastric cancer and highlight new advancements in the treatment of this aggressive cancer.

## Background

Gastric cancer is the fifth-most common cancer worldwide, but the third leading cause of cancer death. According to SEER (Surveillance, Epidemiology and End Results) data from 1976 to 2014, the incidence of gastric cancer has halved in the USA, decreasing from 12.21 to 6.72 per 100,000 people [1]. Despite this decrease in incidence, gastric cancer in the USA has a 5-year survival rate of only 32% for all-comers. This is in part because patients’ symptoms often arise only after progression to locally-advanced or metastatic disease, and because there is a lack of screening in most western nations [2]. Of all patients with gastric adenocarcinoma, it is estimated that 26% have single-site metastasis, and 13% have multi-site metastasis, the most common of which are the liver, peritoneum, and lung [3]. Histologically, gastric cancer has traditionally been classified as intestinal or diffuse types, with the latter having a worse prognosis [4]. Poorly differentiated tumors generally confer a worse survival compared to moderately or well differentiated, with a hazard ratio of 1.19 [5]. In the absence of metastatic disease, surgery is the only potentially curative option. For patients with distant metastasis, however, prognosis is extremely poor and remains only 5.2% at 5 years [1].

Here, we report the case of a 63-year-old male who presented with a poorly differentiated gastric adenocarcinoma, underwent a staging laparoscopy with peritoneal lavage, received neoadjuvant FLOT chemotherapy, underwent a technically successful R0 resection with D2 lymphadenectomy, and ultimately developed an isolated skeletal muscle metastasis. The case highlights a rare example of a skeletal muscle metastasis of gastric cancer, which occurs in 0.03 to 0.16%. A literature review notes at least 34 cases of reported skeletal metastases, occurring in very diverse areas of the body (Table 1). This rare case is particularly notable because it occurred in the setting of a histologically margin-negative (R0) resection and negative lymph nodes on final pathology [37]. The case also provides narrative of the oncologic and surgical management of gastric cancer, with review of prognostic factors.

**Table 1:** Literature cases of skeletal metastases of gastric cancer

	Year	Authors	Age (years)	Sex	Affected muscles
1	1962	Sato et al. [6]	N/A	N/A	Iliopsoas m.
2	1979	Treves and Barruch [7]	52	M	Psoas m.
3	1983	Obley et al. [8]	54	M	Paraspinal m.
4	1983	Fujiwara et al. [9]	74	F	NA
5	1984	Rosenbaum et al. [10]	54	M	Upper arm m., Femoral m.
6	1989	Arnold et al. [11]	59	F	Extraocular m.
7	1990	Porile et al. [12]	65	M	Sartorius m., Rectus femoris m.
8	1993	Sudo et al. [13]	61	M	Trapezius m.
9	1993	Fred et al. [14]	47	F	Extraocular m.
10	1994	Toillon et al. [15]	58	M	Gastrocnemius m.
11	1996	Amano and Kumazaki [16]	57	M	Gastrocnemius m.
12	1997	Ferri et al. [17]	N/A	N/A	Masseter m.
13	1998	Narvaez et al. [18]	49	M	Psoas m.
14	1998	Pestalozzi and von Hochstetter [19]	72	F	Gastrocnemius m.
15	1998	Pinto et al. [20]	N/A	N/A	NA
16	2001	Oba et al. [21]	70	M	Lumbar m., iliopsoas m.
17	2002	Kondo et al. [22]	64	F	Gluteus maximus m., Adductor magnus m.
18	2003	Varma et al. [23]	72	M	Anterior fermoral m.
19	2004	Tuoheti et al. [24]	48	M	Shoulder muscle.
20	2004	Tuoheti et al. [24]	89	M	Gluteal muscle.
21	2006	Bese et al. [25]	60	M	Paravertebral m.
22	2008	Souayah et al. [26]	49	M	Lateral rectus m.
23	2009	Tougeron et al. [27]	71	M	Deltoid m.
24	2011	Sakuma et al. [28]	64	F	Gluteal m.
25	2012	Gogou et al. [29]	N/A	N/A	Femoral m.
26	2014	Pergolini et al. [30]	67	M	Adductor m.
27	2014	Lourenço et al. [31]	68	M	Upper thigh m.
28	2015	Koga et al. [32]	71	M	Multiple
29	2016	Xiao-Xia Wang [33]	63	M	N/A
30	2016	Ebisui [34]	49	F	Femoral mm.
31	2017	Temido et al. [35]	42	M	Extraocular m.
32	2018	Kamitani et al. [36]	47	M	Latissimus dorsi m.
33	2019	Aguirre et al. [37]	57	F	Multiple
34	2020	Daneti et al. [38]	42	M	Psoas m., Gluteal mm.

## Case presentation

**Fig. 1:**
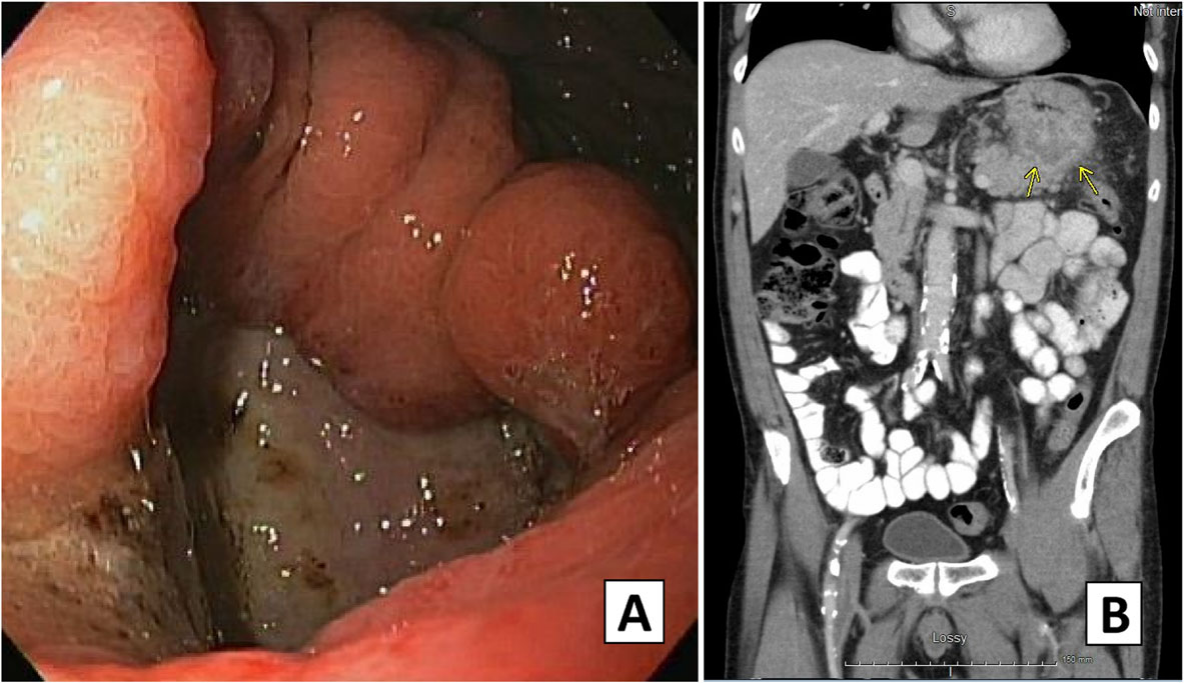
Initial endoscopic appearance of ulcerated mass in gastric fundus (**a**). Preoperative contrast-enhanced CT scan of the abdomen and pelvis (**b**). Yellow arrows demonstrate mass in gastric fundus and body

**Fig. 2:**
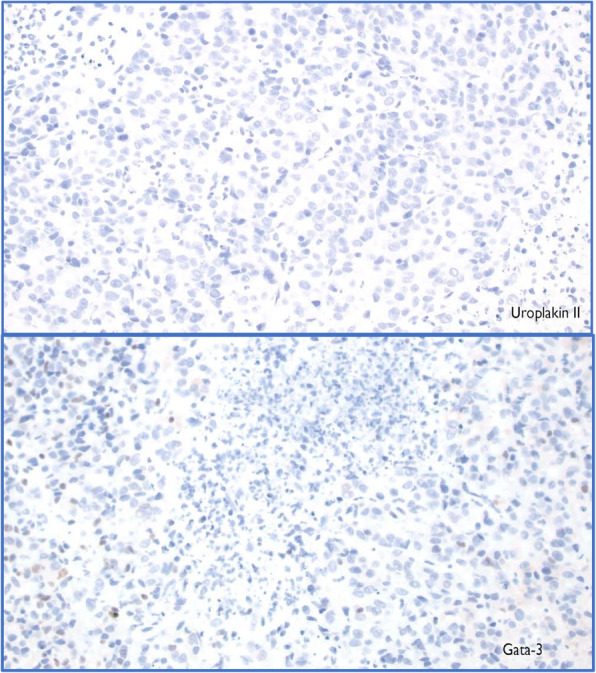
Initial appearance of gastric cancer prior to treatment, staining negative for Uroplakin II and weak for GATA-3

The patient is a 63-year-old man with a history of smoking, COPD, and stage 1 urothelial cancer who presented with a 3-month history of epigastric abdominal pain, early satiety, fatigue, and 12-pound weight loss. Esophagogastroduodenoscopy (EGD) demonstrated a large posterior body gastric ulcer (Fig. 1), and biopsies revealed poorly differentiated adenocarcinoma based on microscopic features. Differential included metastatic urothelial cancer versus a more likely primary gastric cancer. Negative stains for CK20, PSA, PSAP, Uroplakin II, chromogranin, synaptophysin, CK7, and CD56, a weak GATA-3 stain and positive stains for AE1/AE3 confirmed a diagnosis of a new primary gastric cancer (Fig. 2). Endoscopic ultrasound revealed many abnormal lymph nodes in the celiac region (level 20), peripancreatic region, and porta hepatis (largest measuring 9 mm by 5 mm) such that he was staged as a T3N3M0 by EUS criteria. PET scan revealed an FDG avid gastric mass. CT chest, abdomen, and pelvis and PET were negative for distant metastasis (Fig. 1).

Given the presumptive clinical stage T3N3M0, staging laparoscopy with 1 L peritoneal lavage was performed. There was no evidence of any occult peritoneal metastasis at the time of surgery. However, washings were positive for extraluminal mucin, suggestive of a cytology positive lavage. He was presented at our multidisciplinary tumor board conference where the decision was made to proceed with neoadjuvant FLOT chemotherapy followed by restaging. He underwent four cycles of FLOT, followed by a restaging PET CT (positron emission tomography-computed tomography), which was negative for metastatic disease. A second staging laparoscopy with peritoneal washings was performed in the aforementioned fashion and lavage was negative for malignant cells, suggestive of conversion from cytology positive to cytology negative. Repeat EGD and CT imaging showed no significant changes in tumor size. Given the change in his cytological status, the patient was taken to the operating room for a planned gastrectomy, D2 lymphadenectomy, and placement of a feeding jejunostomy tube placement. On exploration of the abdomen, there was no evidence of diffuse metastatic disease. The tumor invaded through the posterior gastric wall and into the pancreatic body and transverse colon. An en bloc resection was performed which included a total gastrectomy, distal pancreatectomy, with splenectomy and transverse colon resection with end colostomy. A stapled Roux-en-Y esophagojejunostomy was constructed and a feeding jejunostomy tube was placed distal to this.

**Fig. 3:**
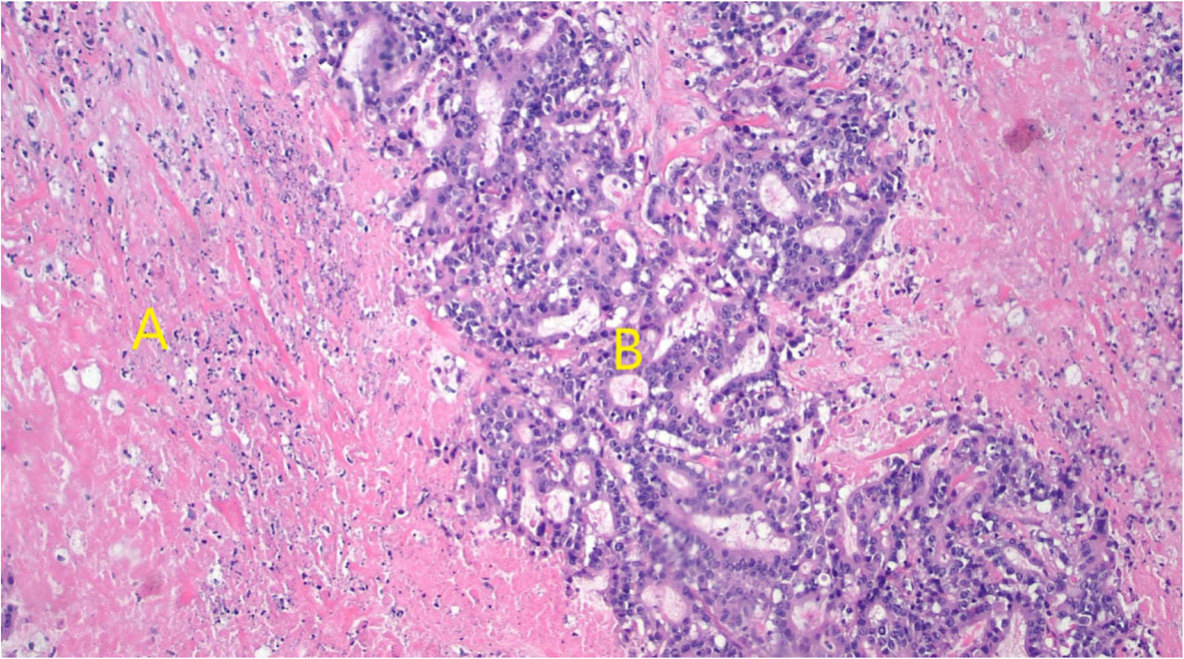
H&E stain showing gastric tumor with necrosis, indicating response to neoadjuvant chemoradiotherapy. **a** Necrotic tumor as evidence of neoadjuvant treatment effect. **b** Viable tumor

Pathology revealed an 8.3 cm, poorly differentiated adenocarcinoma with invasion into the pancreatic parenchyma and histologically negative margins. Thirty-one regional lymph nodes were negative for metastasis making his final stage ypT4b N0 M0, Stage III. His post-operative course was uneventful. There was evidence of treatment effect related necrosis on final pathology indicating response to his neoadjuvant chemotherapy (Fig. 3).

**Fig. 4:**
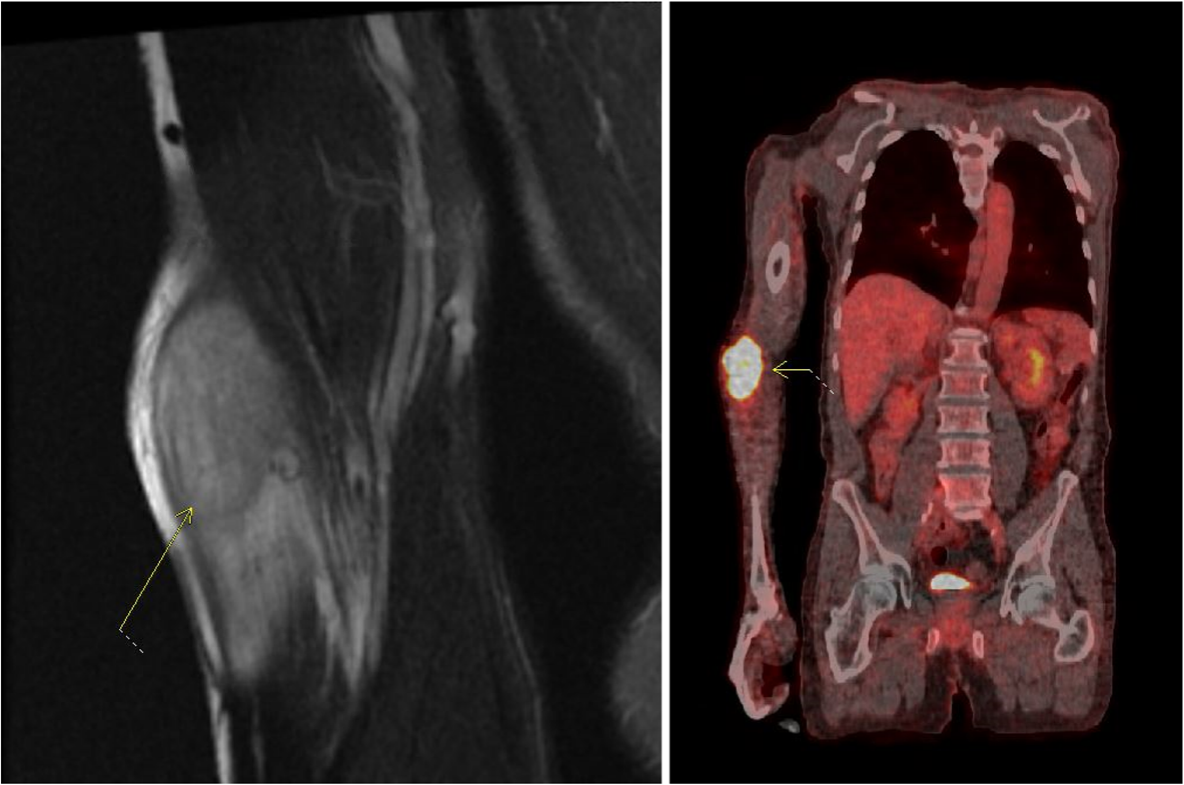
MRI and PET scan imaging of right elbow mass, 4 months post-operatively

**Fig. 5:**
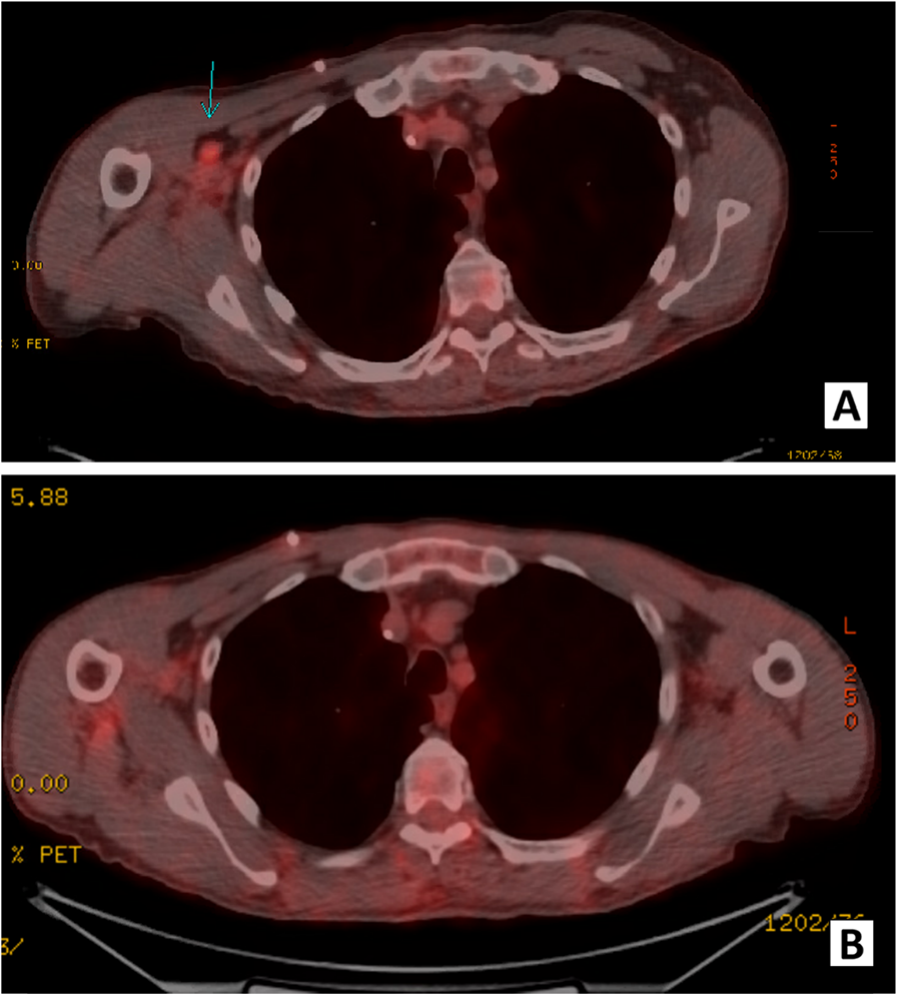
Post-metastasectomy surveillance PET scan demonstrating FDG avid right axillary lymph node (**a**) and resolution of FDG avidity as well as decrease in size of this node (**b**) following immunotherapy, indicating response to immunotherapy. This response persisted on subsequent PET scans and the patient remains disease free at 1 year after diagnosis of metastatic disease

**Fig. 6:**
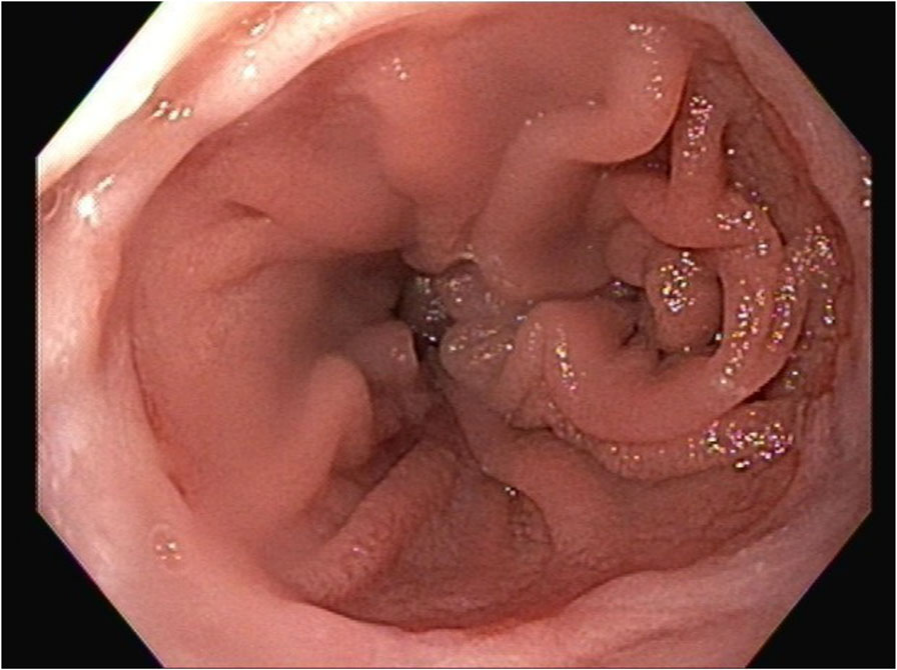
EGD at 11 months after initial resection demonstrating no evidence of local recurrence at the esophagojejunostomy anastomosis

According to NCCN guidelines, surveillance was planned with a history and physical exam every 3–6 months for the first 2 years, and every 6–12 months for the subsequent 3 years, and finally annually thereafter. Surveillance imaging was also planned according to NCCN guidelines with a contrast CT chest, abdomen, and pelvis every 6–12 months for the first 2 years and then annually for 5 years [39]. Approximately 3 months after surgery, the patient developed a rapidly enlarging right lateral forearm mass. MRI revealed a 7-cm heterogeneously enhancing intramuscular mass within the brachioradialis muscle. This mass was found to be FDG avid on PET (SUV 12) and several right axillary lymph nodes were noted to have mild uptake with SUV 2.9 (Fig. 4). Core biopsy revealed poorly differentiated adenocarcinoma, consistent with metastasis from his gastric primary. We discussed with the patient that resection of this mass would not improve his survival and that extremity metastasis has shown to be a poor prognostic sign in the literature [37]. Next generation sequencing was performed on this extremity metastasis, which included a gene profile of at least 500 genes. This was positive for PDL1, suggesting a benefit from immunotherapy. Positive PDL1 was defined by a Combined Positive Score, which is calculated by the number of PDL1 staining cells divided by the total viable tumor cells multiplied by 100. Somatic mutations were also noted in MSH2, MSH6, and PDL1 and were negative in HER2. However, genetic testing revealed no germline mutations such that a diagnosis of Lynch syndrome was not supported. Our recommendation was to initiate systemic therapy with a PDL1 inhibitor (pembroluzimab) prior to resection of this metastasis as a means to evaluate the tumor response to treatment. However, the patient strongly desired upfront resection of the tumor as it was symptomatic, so a successful metastasectomy was performed. Surveillance was continued with a thorough physical examination and PET scan every 3 to 6 months. Surveillance PET scans showed response to immunotherapy with resolution of the FDG avidity in his right axilla, as well as a decrease in the size of the previously FDG avid right axillary node (Fig. 5). EGD performed at 11 months following initial resection did not demonstrate any signs of local tumor recurrence at the esophagojejunostomy anastomosis (Fig. 6). The patient remains disease free at the time of this publication, 20 months from the time of diagnosis and 1 year after the diagnosis of metastatic disease.

## Discussion and conclusions

The incidence of gastric cancer in the United States is 6.72 per 100,000 people, but it remains one of the cancers with the highest mortality. Of those that present with metastasis, the median survival is only three months [3]. Workup typically includes a thorough history and physical exam, EGD and EUS to determine the depth of invasion and evaluate regional lymph nodes, and staging chest/abdomen/pelvic CT with oral and IV contrast. A nutritional assessment is also recommended [39].

The Lauren classification divides gastric adenocarcinoma into intestinal, diffuse (signet ring), and intermediate types. Intestinal type gastric cancer has been shown to have a better 5-year overall survival than diffuse type and mixed type [40]. Gastric cancer is also characterized by the presence of somatic mutations, which develop as the tumor replicates and grows, as well as germline mutations. CDH1 mutations have now been identified in 64% of diffuse types of gastric cancer [41]. CDH1 codes for the E-cadherin, a cell surface protein important in maintaining intercellular connections. Thus, in those with documented CDH1 mutations, a prophylactic total gastrectomy after age 20 may be considered. In patients with diffuse type gastric cancer who do not have CDH1 mutations, germline mutations in PALB2, BRCA1, and RAD51C have been noted [42]. Mutations noted in intestinal-type gastric cancers include TP53, TP73, APC (adenomatous polyposis coli), TFF (trefoil factor family), DCC (deleted in colon cancer), and FHIT (fragile histidine triad) [43–49]. These mutations often affect a given patient’s response to chemotherapy [41]. Of note, the presented patient was positive for mutations in EBV (Epstein-Barr virus), which is typically associated with a favorable prognosis [50].

In the past, the role of perioperative chemotherapy in the management of gastric cancer was of significant focus. This initially entailed perioperative epirubicin, cisplatin, and continuous infusion of 5-flourouracil (ECF) as demonstrated in the seminal MAGIC trial [51], and was later replaced by the perioperative regimen of 5-flourouracil, leucovorin, oxaliplatin, docetaxel (FLOT) due to the improvement in overall survival in those undergoing FLOT [52]. Recently, however, the discovery of targetable mutations unique to a given tumor’s biology has guided chemotherapeutic and immunotherapeutic options. These mutations include PDL-1 as studied in the KEYNOTE trial [53], microsatellite instability (MSI) which appears to predict higher response rate to PDL-1 blocking immunotherapies [54], and HER2 expression against which Trastuzumab can offer benefit [55]. Per national guidelines, immunotherapy is recommended in those with such targetable mutations who have unresectable locally advanced, recurrent or metastatic disease [39]. Our patient expressed both PD-L1 and MSI-H such that postoperative pembrolizumab was initiated with good response. Notably, he also had somatic mutations in MSH2, MSH6, and PDL1 which were expressed in the tumor, but germline testing revealed no such mutations. One limitation of our case report is that NGS testing was performed on the skeletal metastasis and not the primary gastric cancer, so it is difficult to say whether these mutations were present in the primary tumor.

Surgery is the only curative option for gastric cancer patients without metastatic disease. Since CT imaging determines metastatic disease approximately 81% of the time, recent practice guidelines have advocated diagnostic laparoscopy with peritoneal washing to detect metastatic disease in those with cT3 and/or cN+ disease and to help detect occult peritoneal metastases guide management in higher risk patients, particularly when a neoadjuvant course is pursued [56]. For those undergoing washings, cytology positivity is often the strongest predictor gastric-cancer related death on multivariate analysis [57]. However, positive cytology—as in this case report—is also an opportunity to assess chemotherapy response and candidacy for resection. A study of 1241 patients at Memorial Sloan Kettering revealed 93 patients with occult peritoneal metastasis. This study identified a subset of these patients (29%) with improved disease specific survival who were converted from positive to negative cytology [58]. Another trial, in Japan, demonstrated a three-year survival of 76% for this same specific subset of patients who undergo gastrectomy after successful positive-to-negative conversion by neoadjuvant therapy [59]. At the time of curative-intent gastrectomy, the literature clearly favors a more extensive nodal harvest to include hepatic, left gastric, celiac, and splenic arterial lymph nodes as part of what is known as a D2 lymphadenectomy [60]. Clinical trials have demonstrated lower locoregional recurrence and lower gastric-cancer-related death rates in patients who underwent D2 lymphadenectomy than patients who underwent D1 lymphadenectomy [61]. It should be noted that D3 lymphadenectomy is performed in Western societies less frequently than D2 lymphadenectomy owing to the increased morbidity of these extensive D3 nodal dissections with limited benefit in overall survival. Many Western randomized trials have failed to demonstrate a survival advantage with extended nodal dissection and D2 lymphadenectomy is considered to be the standard approach [62]. An adequate lymph node yield for appropriate staging in gastric cancer is considered to be at least 15 nodes, however, multiple studies have demonstrated that higher LN yield may be associated with improved surgical quality and improved overall survival in patients with gastric cancer [63]. At the time of surgery, this patient did not have abnormal portal lymph nodes and decision was made to perform a D2 lymphadenectomy. This operation resulted in a good lymph node yield with 31 nodes, none of which demonstrated any evidence of metastatic disease.

Disease-free survival is related to the adequacy of surgical resection and it is crucial to obtain an R0 resection to decrease rates of local recurrence. Gastric cancer-specific survival 5 years after an R0 resection has been shown to be 50%, while it is only 13–29% with an R1 resection [64–66].

Metastasectomy in gastric cancer is controversial. While some Japanese literature has reported increased survival after radical hepatic resection, this has not been consistently reproducible in other populations [67]. There are multiple pathways of gastric cancer dissemination in metastatic disease: lymphatic dissemination (74–88%), subperitoneal dissemination along the perigastric ligaments, mesentery, or omentum, direct invasion into adjacent organs (i.e., esophagus 60%), transperitoneal seeding (53%), and hematogenous dissemination (i.e., as seen in the rate of hepatic metastases) [68]. Though metastasectomy in gastric cancer has not been shown to improve overall survival, it may be considered as a reasonable palliative option if the patient is symptomatic and if it does not increase patient morbidity, as was the case in this patient.

Only 30 cases of skeletal muscle metastasis after a primary gastric cancer have been reported in the literature since 1960, and skeletal muscle metastasis portends an extremely poor prognosis [37]. This unique case report presents a patient with moderate response to neoadjuvant chemotherapy such that he ultimately underwent an R0 resection, but subsequently developed an isolated skeletal metastasis.

This report highlights that while gastric cancer remains a highly aggressive cancer, survival has significantly improved with neoadjuvant therapy, improvements in surgical technique and the advent of targeted therapies.

## Data Availability

Not applicable.

## Abbreviations

R0: Microscopically margin-negative resection
D2: Lymphadectomy harvesting hepatic, left gastric, celiac, and splenic arterial lymph nodes
SEER: Surveillance, Epidemiology and End Results
FLOT: 5-Flourouracil, leucovorin, oxaliplatin, docetaxel
EGD: Esophagogastroduodenoscopy
EUS: Endoscopic ultrasound
PET-CT: Positron emission tomography-computed tomography
NCCN: National Comprehensive Cancer Network
MRI: Magnetic resonance imaging
FDG: Fluorodeoxyglucose
NGS: Next generation sequencing
MSI: Microsatellite instability

## References

[1] SEER Data (Surveillance E, and End Results). Cancer of the stomach. Cancer Statistics Review (1975-2014) (https://seer.cancer.gov/archive/csr/1975_2014/), National Cancer Institute, DCCPS, Surveillance Research Program, updated April 2, 2018.

[2] Maconi G, Manes G, Porro GB (2008). Role of symptoms in diagnosis and outcome of gastric cancer. World J Gastroenterol.

[3] Riihimaki M, Hemminki A, Sundquist K, Sundquist J, Hemminki K (2016). Metastatic spread in patients with gastric cancer. Oncotarget.

[4] Hu B, El Hajj N, Sittler S, Lammert N, Barnes R, Meloni-Ehrig A (2012). Gastric cancer: classification, histology and application of molecular pathology. J Gastrointest Oncol.

[5] Yang D, Hendifar A, Lenz C, Togawa K, Lenz F, Lurje G, Pohl A, Winder T, Ning Y, Groshen S, Lenz HJ (2011). Survival of metastatic gastric cancer: significance of age, sex and race/ethnicity. J Gastrointest Oncol.

[6] Sato N, Sawae Y, Fukamachi K (1962). Case of stomach cancer with remote metastases to the entire body, especially to the spleen and bilateral iliopsoas. Naika Intern Med.

[7] Treves R, Barruch D, Desproges-Gotteron R. Les metastases musculaires. Sem Hop Paris. 1979;55:1471–5. 230594

[8] Obley DL, Slasky BS, Peel RL, Rosenbaum LH, Nicholas JJ, Ellis LD (1983). Bone-forming gastric metastases in muscle—computed tomographic demonstration. J Comput Tomogr.

[9] Fujiwara R, Saga T, Akashi N, Takegoshi T, Saitoh K, Tokuda T, Kitoh C, Ichiyanagi K, Doishita K (1983). Heterotopic ossification in the skeletal muscle metastases of advanced stomach cancer. Gan no rinsho Jpn J Cancer Clin.

[10] Rosenbaum LH, Nicholas JJ, Slasky BS, Obley DL, Ellis LD (1984). Malignant myositis ossificans: occult gastric carcinoma presenting as an acute rheumatic disorder. Ann Rheum Dis.

[11] Arnold RW, Adams BA, Camoriano JK, Dyer JA (1989). Acquired divergent strabismus: presumed metastatic gastric carcinoma to the medial rectus muscle. J Pediatr Ophthalmol Strabismus.

[12] Porile JL, Olopade OI, Hoffman PC (1990). Gastric adenocarcinoma presenting with soft tissue masses. Am J Gastroenterol (Springer Nature).

[13] Sudo AK, Ogihara YO, Shiokawa YA, Fujinami SH, Sekiguchi SH (1993). Intramuscular metastasis of carcinoma. Clin Orthop Relat Res.

[14] Fred W, Van Gelderen C (1993). Gastric carcinoma metastases to extraocular muscles. J Comput Assist Tomogr.

[15] Toillon M, Lepage M, Naudin P, Moreau M, Trutaud-Muresan A, Lamotte A (1994). Muscular metastasis from a gastric adenocarcinoma. Gastroenterol Clin Biol.

[16] Amano Y, Kumazaki T (1996). Gastric carcinoma metastasis to calf muscles: MR findings. Radiat Med.

[17] Ferri J, Vandenhaute B, Donazzan M (1997). Intra-masseter metastasis of a gastric adenocarcinoma. Rev Stomatol Chir Maxillofac.

[18] Narvaez JA, Narvaez J, Clavaguera MT, Juanola X, Valls C, Fiter J (1998). Bone and skeletal muscle metastases from gastric adenocarcinoma: unusual radiographic, CT and scintigraphic features. Eur Radiol.

[19] Pestalozzi BC, Von Hochstetter AR (1998). Muscle metastasis as initial manifestation of adenocarcinoma of the stomach. Schweiz Med Wochenschr.

[20] Pinto F, Falleni A, Campoccia S, Lischi DM (1998). Muscular metastasis of a gastric carcinoma: the first sign of a recurrence of the disease. A case. Radiol Med.

[21] Oba K, Ito T, Nakatani C, Okamura K, Yamaguchi H, Ajiro Y, Suzuki T, Nakano H, Metori S, Sano K, Hyakusoku H (2001). An elderly patient with gastric carcinoma developing multiple metastasis in skeletal muscle. J Nippon Med Sch.

[22] Kondo S, Onodera H, Kan S, Uchida S, Toguchida J, Imamura M (2002). Intramuscular metastasis from gastric cancer. Gastric Cancer.

[23] Varma GN, Winston JS, Hill HC, Gibbs JF, Proulx GM (2003). Case 3. Gastric signet ring carcinoma presenting as a diffuse thigh mass. J Clin Oncol.

[24] Tuoheti Y, Okada K, Osanai T, Nishida J, Ehara S, Hashimoto M, Itoi E (2004). Skeletal muscle metastases of carcinoma: a clinicopathological study of 12 cases. Jpn J Clin Oncol.

[25] Beşe NŞ, Özgüroĝlu M, Dervişoĝlu S, Kanberoglu K, Öber A (2006). Skeletal muscle: an unusual site of distant metastasis in gastric carcinoma. Radiat Med.

[26] Souayah N, Krivitskaya N, Lee HJ (2008). Lateral rectus muscle metastasis as the initial manifestation of gastric cancer. J Neuroophthalmol.

[27] Tougeron D, Hamidou H, Dujardin F, Maillard C, Di Fiore F, Michel P (2009). Unusual skeletal muscle metastasis from gastric adenocarcinoma. Gastroenterol Clin Biol.

[28] Sakuma T, Deguchi R, Takashimizu S, Ogasawara F, Numata M, Ohtani Y, Sato S, Mine T, Iwata Y (2011). Good response chemotherapy for late-recurring gastric cancer in the gluteals, with peritoneal and retroperitoneal dissemination. Tokai J Exp Clin Med.

[29] Gogou PV, Polydorou A, Papacharalampous XN, Kondi-Paphiti A, Balafouta MJ, Gennatas CS, Kouvaris JR (2012). Femoral muscle metastasis from gastric carcinoma. Turk J Gastroenterol.

[30] Pergolini I, Crippa S, Santinelli A, Marmorale C (2014). Skeletal muscle metastases as initial presentation of gastric carcinoma. Am J Case Rep.

[31] Lourenço LG, Carlotto JR, Herbella FA, Silva DA, Setti HB (2014). Muscular metastasis from gastric cancer. J Gastrointest Oncol.

[32] Koga Y, Baba Y, Harada K, Kosumi K, Shigaki H, Kurashige J, Ishimoto T, Iwatsuki M, Miyamoto Y, Sakamoto Y, Yoshida N (2015). Multiple skeletal muscle metastases from poorly differentiated gastric adenocarcinoma. Surg Case Rep.

[33] Wang XX, Liu HQ, Deng FR, Liu J (2016). Skeletal muscle metastases as the first sign of a recurrence of gastric carcinoma. Dig Liver Dis.

[34] Ebisui C, Hamano R, Nushijima Y, Yanagisawa T, Okamura S, Fukuchi N, Murata K, Yokouchi H, Kinuta M, Ohishi K (2016). A case of T1a early gastric cancer that metastasized to the right femoral muscles six years and seven months after radical surgery. Gan to kagaku ryoho Cancer Chemother.

[35] Temido H, Vilão F, Parente F (2017). Diplopia as rare initial manifestation of gastric cancer. Acta Med Port.

[36] Kamitani N, Watanabe A, Kirihataya Y, Ko S (2018). Metachronous skeletal muscle metastasis without any other organ metastases after curative gastrectomy: a case report. Surg Case Rep.

[37] Aguirre LE, Salcedo J, Zuquello R, Garcia-Buitrago M, Ardalan B (2019). Metastatic involvement of skeletal muscle from gastric adenocarcinoma. Oxf Med Case Rep.

[38] Daneti D, Senthamizhselvan K, Verma SK, Mohan P (2021). Gastric adenocarcinoma presenting with multiple skeletal muscle metastases. BMJ Case Rep.

[39] Ajani JAM, D’Amico TA, Bentrem DJ, Chao J, Corvera C, Das P, Denlinger CS, Enzinger PC, Fanta P, Farjah F, Gerdes H, Gibson M, Glasgow RE, Hochwald S, Hofstetter WL, Ilson DH, Johung KL, Keswani RN, Kleinberg LR, Klempner S, Leong S, Ly QP, Matkovskyj KA, McNamara M, Mucahy MF, Paluri RK, Park H, Perry KA, Pimiento J, Poultsides GA, Roses RE, Strong VE, Wiesner G, Willett CG, Yakoub D (2020). National Comprehensive Cancer Network (NCCN) clinical practice guidelines in oncology: gastric cancer.

[40] Chen YC, Fang WL, Wang RF, Liu CA, Yang MH, Lo SS, Wu CW, Li AFY, Shyr YM, Huang KH (2016). Clinicopathological variation of lauren classification in gastric cancer. Pathol Oncol Res.

[41] Tan IB, Ivanova T, Lim KH, Ong CW, Deng N, Lee J (2011). Intrinsic subtypes of gastric cancer, based on gene expression pattern, predict survival and respond differently to chemotherapy. Gastroenterology.

[42] Sahasrabudhe R, Lott P, Bohorquez M, Toal T, Estrada AP, Suarez JJ, Brea-Fernández A, Cameselle-Teijeiro J, Pinto C, Ramos I, Mantilla A, Prieto R, Corvalan A, Norero E, Alvarez C, Tapia T, Carvallo P, Gonzalez LM, Cock-Rada A, Solano A, Neffa F, Della Valle A, Yau C, Soares G, Borowsky A, Hu N, He LJ, Han XY, Taylor PR, Goldstein AM, Torres J, Echeverry M, Ruiz-Ponte C, Teixeira MR, Carvajal-Carmona LG, Echeverry M, Bohorquez M, Prieto R, Suarez J, Mateus G, Bravo MM, Bolaños F, Vélez A, Corvalan A, Carvallo P, Torres J, Carvajal-Carmona L (2017). Germline mutations in PALB2, BRCA1, and RAD51C, which regulate DNA recombination repair, in patients with gastric cancer. Gastroenterology.

[43] Xiao YP, Wu DY, Xu L, Xin Y (2006). Loss of heterozygosity and microsatellite instabilities of fragile histidine triad gene in gastric carcinoma. World J Gastroenterol.

[44] Sato K, Tamura G, Tsuchiya T, Endoh Y, Usuba O, Kimura W, Motoyama T (2001). Frequent loss of expression without sequence mutations of the DCC gene in primary gastric cancer. Br J Cancer.

[45] Yasui W, Sentani K, Motoshita J, Nakayama H (2006). Molecular pathobiology of gastric cancer. Scand J Surg.

[46] Yokozaki H, Shitara Y, Fujimoto J, Hiyama T, Yasui W, Tahara E (1999). Alterations of p73 preferentially occur in gastric adenocarcinomas with foveolar epithelial phenotype. Int J Cancer.

[47] Morgan C, Jenkins GJ, Ashton T, Griffiths AP, Baxter JN, Parry EM (2003). Detection of p53 mutations in precancerous gastric tissue. Br J Cancer.

[48] Shiao YH, Rugge M, Correa P, Lehmann HP, Scheer WD (1994). p53 alteration in gastric precancerous lesions. Am J Pathol.

[49] Tomkova K, Belkhiri A, El-Rifai W, Zaika AI (2004). p73 isoforms can induce T-cell factor-dependent transcription in gastrointestinal cells. Cancer Res.

[50] Liu X, Liu J, Qiu H, Kong P, Chen S, Li W, Zhan Y, Li Y, Chen Y, Zhou Z, Xu D, Sun X (2015). Prognostic significance of Epstein-Barr virus infection in gastric cancer: a meta-analysis. BMC Cancer.

[51] Cunningham D, Allum WH, Stenning SP, Thompson JN, Van de Velde CJ, Nicolson M (2006). Perioperative chemotherapy versus surgery alone for resectable gastroesophageal cancer. N Engl J Med.

[52] Al-Batran SE, Homann N, Pauligk C, Goetze TO, Meiler J, Kasper S (2019). Perioperative chemotherapy with fluorouracil plus leucovorin, oxaliplatin, and docetaxel versus fluorouracil or capecitabine plus cisplatin and epirubicin for locally advanced, resectable gastric or gastro-oesophageal junction adenocarcinoma (FLOT4): a randomised, phase 2/3 trial. Lancet.

[53] Fuchs CS, Doi T, Jang RW, Muro K, Satoh T, Machado M, Sun W, Jalal SI, Shah MA, Metges JP, Garrido M, Golan T, Mandala M, Wainberg ZA, Catenacci DV, Ohtsu A, Shitara K, Geva R, Bleeker J, Ko AH, Ku G, Philip P, Enzinger PC, Bang YJ, Levitan D, Wang J, Rosales M, Dalal RP, Yoon HH (2018). Safety and efficacy of pembrolizumab monotherapy in patients with previously treated advanced gastric and gastroesophageal junction cancer: phase 2 clinical KEYNOTE-059 trial. JAMA Oncol.

[54] Brar G, Shah MA (2019). The role of pembrolizumab in the treatment of PD-L1 expressing gastric and gastroesophageal junction adenocarcinoma. Therap Adv Gastroenterol.

[55] Apicella M, Corso S, Giordano S (2017). Targeted therapies for gastric cancer: failures and hopes from clinical trials. Oncotarget.

[56] Coburn N, Cosby R, Klein L, Knight G, Malthaner R, Mamazza J, Mercer CD, Ringash J (2017). Staging and surgical approaches in gastric cancer: a clinical practice guideline. Curr Oncol.

[57] De Andrade JP, Mezhir JJ (2014). The critical role of peritoneal cytology in the staging of gastric cancer: an evidence-based review. J Surg Oncol.

[58] Mezhir JJ, Shah MA, Jacks LM, Brennan MF, Coit DG, Strong VE (2011). Positive peritoneal cytology in patients with gastric cancer: natural history and outcome of 291 patients. Indian J Surg Oncol.

[59] Yasufuku I, Nunobe S, Ida S, Kumagai K, Ohashi M, Hiki N, Sano T (2020). Conversion therapy for peritoneal lavage cytology-positive type 4 and large type 3 gastric cancer patients selected as candidates for R0 resection by diagnostic staging laparoscopy. Gastric Cancer.

[60] Schmidt B, Yoon SS (2013). D1 versus D2 lymphadenectomy for gastric cancer. J Surg Oncol.

[61] Songun I, Putter H, Kranenbarg EM, Sasako M, van de Velde CJ (2010). Surgical treatment of gastric cancer: 15-year follow-up results of the randomised nationwide Dutch D1D2 trial. Lancet Oncol.

[62] Douridas GN, Pierrakakis SK (2018). Is there any role for D3 lymphadenectomy in gastric cancer?. Front Surg.

[63] Samples JE, Stitzenberg KB, Meyers MO (2014). Lymph node yield and survival in gastric carcinoma. JCO.

[64] Liang Y, Ding X, Wang X, Wang B, Deng J, Zhang L, Liang H (2015). Prognostic value of surgical margin status in gastric cancer patients. ANZ J Surg.

[65] Morgagni P, Garcea D, Marrelli D, De Manzoni G, Natalini G, Kurihara H (2008). Resection line involvement after gastric cancer surgery: clinical outcome in nonsurgically retreated patients. World J Surg.

[66] Kattan MW, Karpeh MS, Mazumdar M, Brennan MF (2003). Postoperative nomogram for disease-specific survival after an R0 resection for gastric carcinoma. J Clin Oncol.

[67] Miyazaki M, Itoh H, Nakagawa K, Ambiru S, Shimizu H, Togawa A, Shiobara M, Ohtsuka M, Sasada K, Shimizu Y, Yoshioka S, Nakajima N, Suwa T, Kimura F. Hepatic resection of liver metastases from gastric carcinoma. Am J Gastroenterol. 1997;92(3):490–3. 9068476

[68] Young JJ, Pahwa A, Patel M, Jude CM, Nguyen M, Deshmukh M, Huang L, Mohammad SF. Ligaments and lymphatic pathways in gastric adenocarcinoma. Radiographics. 2019;39(3):668–89. 10.1148/rg.2019180113. 10.1148/rg.2019180113 30951438

